# Supplementary material for: Prehabilitation Prior to Chemotherapy in Humans: A Review of Current Evidence and Future Directions
Source: Cancers (Basel). 2025 Aug 15;17(16):2670. doi: 10.3390/cancers17162670 (PMC12384665; doi:10.3390/cancers17162670)
Supplement: Supplementary file 1 [file cancers-17-02670-s001.zip › cancers-3781161-supplementary.pdf]

# Supplementary Materials: Prehabilitation Prior to Chemotherapy in Humans: A Review of Current Evidence and Future Directions

**Karolina Pietrakiewicz, Rafał Stec and Jacek Sobocki**

**Table S1.** Proposal for multidisciplinary prehabilitation protocol (potential guidelines for future research).

<table>
<tr><th>Areas of interventions</th><th colspan="2">Key components</th><th>Recommendations</th><th>Additions</th></tr>
<tr><td rowspan="6">Diet and pharmaconutrients</td><td colspan="2">Protein</td><td>Modification of the oral diet by a dietitian; optional consideration of introducing oral nutritional supplements as a complement to the oral diet, if required by the patient's condition; 1.4 – 1.6 g/kg of body mass sourced on whey, distributed evenly in each meal throughout a day</td><td>Whey is preferred over casein due to its higher bioavailability</td></tr>
<tr><td colspan="2">Omega-3 fatty acids</td><td>Fish supplement containing EPA + DHA at a ratio lower than 2:1</td><td>EPA up to 5 g/day is safe;<br>effectiveness is observed while supplementing approximately 2.2 g of EPA/day</td></tr>
<tr><td rowspan="4">Vitamins</td><td>D</td><td>Correction of vitamin D serum level when lower than 30 ng/ml</td><td rowspan="4">Not applicable</td></tr>
<tr><td>E</td><td>Vitamin E supplementation is not recommended unless deficiency is confirmed and not possible to correct via modifications in diet</td></tr>
<tr><td>A</td><td>Vitamin A supplementation is not recommended unless deficiency is confirmed and not possible to correct via modifications in diet</td></tr>
<tr><td>C</td><td>In breast cancer, use of vitamin C can be considered</td></tr>
<tr><td rowspan="2">Physical activity</td><td colspan="2">Aerobic exercises</td><td>Three times per week/ minimum 35 minutes for 12 weeks most effectively</td><td rowspan="2">There is a potential for reducing cardiotoxicity and improving $VO_2max$;<br>supervised by a physician<br>followed by the FIIT principles</td></tr>
<tr><td colspan="2">Strength training</td><td>Two times per week/ minimum 30 minutes for 12 weeks most effectively</td></tr>
<tr><td>Counselling</td><td colspan="2">Clinical psychologists, psycho-oncologists</td><td>Psychological support led by a specialist is key after the diagnosis</td><td>Physical activity is linked to better mental well-being</td></tr>
<tr><td>Smoking cessation</td><td colspan="2">Nicotine</td><td>Nicotine cessation is favorable, combining behavioral and pharmacological interventions seem to be the most effective</td><td>Not applicable</td></tr>
</table>

EPA—eicosapentaenoic acid; DHA—docosahexaenoic acid; $VO_2max$—maximal oxygen uptake; FITT—frequency, intensity, time, and type.
